# Cesarean delivery was associated with low morbidity in very low birth weight infants: A retrospective cohort study

**DOI:** 10.1097/MD.0000000000033554

**Published:** 2023-04-21

**Authors:** Jianqiong Li, Tingting Zhu, Gu Liu, Yuyang Chen, Linli Xing, Yichao Tian, Fengbing Liang

**Affiliations:** a Department of Obstetrics and Gynecology, Sir Run Run Shaw Hospital, School of Medicine, Zhejiang University, Hangzhou, China; b Key Laboratory of Reproductive Dysfunction Management of Zhejiang Province, School of Medicine, Zhejiang University, Hangzhou, China; c Department of Neonatology, Sir Run Run Shaw Hospital, School of Medicine, Zhejiang University, Hangzhou, China; d Hangzhou Xixi Hospital, Zhejiang University School of Medicine, Hangzhou, China.

**Keywords:** cesarean delivery, neonatal morbidity, neonatal mortality, vaginal delivery, very low birth weight

## Abstract

To estimate the relationship among the cesarean delivery (CD), mortality and morbidity in very low birth weight (VLBW) infants weighing less than 1500 g. This retrospective cohort study enrolled 242 VLBW infants delivered between the 24 to 31week of gestation from 2015 to 2021. We compared CD with vaginal delivery (VD). The primary outcome was a composite neonatal morbidity including bronchopulmonary dysplasia, intraventricular hemorrhage, periventricular leukomalacia, necrotizing enterocolitis, late-onset sepsis and retinopathy of prematurity. The secondary outcome included mortality within 28 days. A multivariate logistic regression was used and adjusted for birthweight, twin pregnancy and antenatal steroids intake. The overall CD rate was 80.6%. Compared with VD, a significantly lower composite neonatal morbidity was associated with CD (adjusted odds ratio, 0.33, 95% confidence interval, 0.12–0.90, *P* = .031). The relationship between CD and neonatal morbidity disappeared when the VLBW infants were stratified according to the gestational age. No significant difference was observed between the VD and CD cohorts regarding mortality. Compared with VD, CD was associated with a lower morbidity in VLBW infants. Further studies are required to clarify how this association is influenced by gestational age.

## 1. Introduction

Over the past decades, the worldwide improvements in perinatal and neonatal care have contributed to a better prognosis in very low birth weight (VLBW) infants,^[1–3]^ whose birthweight at delivery were less than 1500 g, as well as resulted in earlier feasible gestational ages.^[4–6]^ However, the optimal mode of delivery for VLBW infants remains a matter of debate.^[6–10]^

In China, preterm birth is defined at ≥ 28 weeks, which means that the parents of infants under 28 gestational weeks can refuse medical treatment for these infants, in consideration of expensive medical expenses and poor prognoses. Not only the parents but also obstetricians and pediatricians in China were pessimistic about infants with less than 28 gestational weeks several years ago owing to insufficient treatment experience. For the past few years, Chinese obstetricians and pediatricians have committed to reduce the morbidity and mortality of preterm infants, drawing on the experiences of developed countries.^[11,12]^ There is more attention towards the proper medical management of VLBW infants in China. The optimal mode of delivery for VLBW infants have been studied in developed countries but relevant data in China are lacking and may be different taking into account policy implications and differences in neonatal care capacity.

The incidence of cesarean delivery (CD) in VLBW infants has increased exponentially worldwide,^[2]^ as well as in China, making difficult to perform randomized controlled trials to investigate the optimal mode of delivery for VLBW infants.^[13]^ In addition, the obvious ethical issues cannot be ignored. Therefore, considering the above factors, we conducted this single-center retrospective cohort study, in which we analyzed whether CD ameliorated morbidity and/or mortality in VLBW infants.

## 2. Materials and methods

### 2.1. Patients

This retrospective cohort study enrolled patients who delivered infants between 24 and 31weeks of gestation from 2015 to 2021. Patients were identified and recruited at the Sir Run Run Shaw Hospital, School of Medicine, Zhejiang University (China) after a detailed review of the electronic medical records. Inclusion criterion comprised the birthweight between 500 and 1500 g. Exclusion criteria consisted of triple pregnancy, intrauterine fetal demise and refusal of medical treatment.

Maternal data collection included maternal age at the delivery, parity, body mass index, singleton pregnancy or twin pregnancy, assisted reproduction, gestational diabetes mellitus and pregnancy-induced hypertension.

Obstetrical and neonatal data included gestational age at the delivery, mode of delivery, gender and birthweight of the infant, 5-minutes Apgar score, antenatal steroids intake and presentation of fetus at the delivery (vertex or nonvertex).

The primary outcome was the composite neonatal morbidity, including bronchopulmonary dysplasia,^[14]^ intraventricular hemorrhage,^[15]^ periventricular leukomalacia,^[16]^ necrotizing enterocolitis,^[17]^ late-onset sepsis,^[18]^ and retinopathy of prematurity.^[19]^ We followed up the preterm infants to their term-equivalent age. These data were obtained from the neonatal charts and confirmed by the neonatology physician. The secondary outcome included mortality within 28 days. Gestational age was specified by measurement of the last available menstrual period and the first trimester ultrasonogram.^[20]^

The Ethics Committee of the Sir Run Run Shaw Hospital reviewed and approved the study. All procedures were performed in conformity to the Helsinki Declaration and its later amendments. The written informed consent was not obtained due to the retrospective nature of the study. Prior to analysis, the patients´ records were anonymized.

### 2.2. Sample size

Sample size was calculated using the following formula.


$$\begin{array}{l} n=\frac{{\left[z_{\alpha }\sqrt{2pq}+z_{\beta }\sqrt{p_{1}\left(1-p_{1}\right)+p_{2}\left(1-p_{2}\right)}\right]}^{2}}{{\left(p_{1}-p_{2}\right)}^{2}} \end{array}$$

Combining the literature reports and our pilot study, the composite neonatal morbidity for cesarean delivery was 60%, and for vaginal delivery, it was 80%. With a significance level (alpha) of 0.05, a power of 80%, and a two-sided test, the calculated sample size for this study was 164.

### 2.3. Statistical analysis

Continuous data without a normal distribution are presented as median with interquartile range. Categorical data are presented as frequencies (%). Continuous variables without a normal distribution were compared by using the Wilcoxon-Mann–Whitney test. Categorical variables were compared by using the chi-square or Fisher’s exact test.

A logistic regression model investigated the association among CD, mortalities and morbidities of VLBW infants. We selected potential confounders influencing the outcome. After adjusting for birthweight, twin pregnancy and antenatal steroids intake, the odds ratios with 95% confidence interval were estimated with a multiple logistic regression.

Secondary analysis of mortality and morbidity was performed in subgroups defined by gestational ages (24–27 and 28–31 weeks of gestation). A *P* value < .05 was considered statistically significant. Data were analyzed by SPSS version 26.0 (STATA Corp., College, TX).

## 3. Results

### 3.1. Cesarean delivery rate

**Figure 1.**
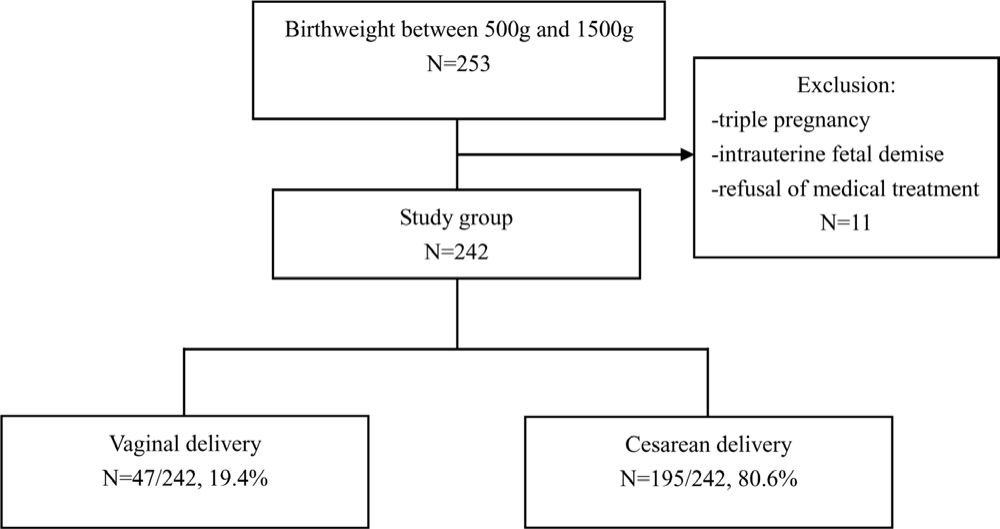
The flowchart of the study.

The study cohort consisted of 242 deliveries. Among them, 195 (80.6%) were CDs and 47 (19.4%) were vaginal deliveries (VDs). When the VLBW infants were stratified in 2 subgroups based on the gestational age (24–27 and 28–31 weeks of gestation), the CD rates were 71.7% and 87.5%, respectively (Fig. 1).

### 3.2. Demographic and perinatal characteristics

According to the mode of delivery, demographic and perinatal characteristics of VLBW infants are shown in Table 1. Compared with the VD cohort, the CD cohort reported more twin pregnancies and more infants with an antenatal steroids intake.

**Table 1:** Demographic and perinatal characteristics according to the mode of delivery.

Characteristics	Vaginal delivery	Cesarean section	*P* value
Maternal age ≥ 35 yr, n (%)	13 (28.9)	44 (22.3)	.35
BMI ≥ 30 kg/m^2^, n (%)	8 (17.8)	20 (10.2)	.150
Nulliparous, n (%)	33 (73.3)	153 (77.7)	.53
Twin pregnancy, n (%)	17 (37.8)	107 (54.3)	.05
Premature rupture of membranes, n (%)	21 (46.7)	104 (52.8)	.46
Gestational diabetes mellitus, n (%)	6 (13.3)	21 (10.7)	.61
Pregnancy induced hypertension syndrome, n (%)	2 (4.4)	22 (11.2)	.27
Antenatal steroids, n (%)	37 (82.2)	183 (92.9)	.04*
Placental abruption, n (%)	2 (4.4)	16 (8.1)	.54
Gestational age at delivery			
24–25 wk, n (%)	11 (24.4)	4 (2.0)	
26–27 wk, n (%)	17 (37.8)	74 (37.6)	
28–29 wk, n (%)	12 (26.7)	78 (39.6)	
30–31 wk, n (%)	5 (11.1)	41 (20.8)	<.001*
Birth weight, M (IQR)	1030 (483)	1110 (395)	.09
Fetal gender (male), n (%)	29 (64.4)	97 (49.20)	.07
Apgar score < 5 at 5 min, n (%)	8 (17.8)	22 (11.2)	.26
Presentation at the time of delivery			
Vertex, n (%)	35 (77.8)	139 (70.6)	
Nonvertex, n (%)	10 (22.2)	58 (29.4)	.33

BMI = body mass index, IQR = interquartile range, M = median.

* *P* < .05 compared with vaginal delivery.

### 3.3. Mortality and morbidity according to the mode of delivery

The mortality and morbidity according to the mode of delivery are shown in Table 2. A neonatal morbidity was described in 164 out of 242 (67.8%) deliveries, while the mortality within 28 days occurred in 23 out of 242 (9.5%) deliveries. After adjusting for birthweight, twin pregnancy and antenatal steroids intake, CD was associated with a lower composite neonatal morbidity compared to VD (adjusted OR, 0.33, 95% CI, 0.12–0.90, *P =* .031). When stratified in subgroups, demographic and perinatal characteristics of VLBW infants are shown in Table 3. And there was no difference between VD and CD cohorts concerning the composite neonatal morbidity (Table 4). Regarding mortality, no difference was observed between VD and CD cohorts.

**Table 2:** Single and composite morbidity and mortality based on the mode of delivery.

Morbidity and mortality	Vaginal delivery (n = 47), n (%)	Cesarean section (n = 195), n (%)	Unadjusted OR (95% CI)	Adjusted OR (95% CI)	*P* value
Composite neonatal morbidity	38 (84.4)	126 (64.0)	0.33 (0.14–0.77)	0.33 (0.12–0.90)	.03
Bronchopulmonary dysplasia	23 (51.1)	73 (37.1)	0.56 (0.29–1.08)	0.70 (0.35–1.42)	.33
Intraventricular hemorrhage	2 (4.4)	13 (6.6)	1.51 (0.33–6.98)	1.70 (0.34–8.51)	.52
Periventricular leukomalacia	1 (2.2)	2 (0.5)	0.22 (0.14–3.66)	0.19 (0.01–4.01)	.29
Necrotizing enterocolitis	6 (13.3)	28 (14.2)	1.01 (0.42–2.78)	1.04 (0.37–2.91)	.94
Late-onset sepsis	7 (15.6)	22 (11.2)	0.68 (0.27–1.71)	0.72 (0.27–1.93)	.52
Retinopathy of prematurity	20 (44.4)	61 (31.0)	0.56 (0.29–1.09)	0.62 (0.30–1.29)	.20
Death within 28 d of delivery	9 (20.0)	14 (7.1)	0.31 (0.12–0.76)	0.57 (0.17–1.90)	.36

CI = confidence interval, OR = odds ratio.

**Table 3:** Demographic and perinatal characteristics in gestational age subgroups according to the mode of delivery.

Characteristics	24–27 weeks			28–31 weeks			
	Vaginal delivery	Cesarean delivery	*P* value		Vaginal delivery	Cesarean delivery	*P* value
Maternal age ≥ 35 yr, n (%)	8 (28.6)	23 (29.5)	.93		5 (29.4)	21 (17.6)	.41
BMI ≥ 30 kg/m^2^, n (%)	8 (28.6)	9 (11.5)	0.07		0 (0)	11 (9.2%)	.41
Nulliparous, n (%)	19 (67.9)	59 (75.6)	.42		14 (82.4)	94 (79.0)	.75
Twin pregnancy, n (%)	11 (39.3)	49 (62.8)	.03*		6 (35.6)	58 (48.7)	.30
Premature rupture of membranes, n (%)	11 (39.3)	43 (55.1)	.15		10 (58.8)	61 (51.3)	.56
Gestational diabetes mellitus, n (%)	2 (7.1)	2 (2.6)	.61		4 (23.5)	19 (16.0)	.67
Pregnancy induced hypertension syndrome, n (%)	0 (0)	9 (11.5)	.11		2 (11.8)	13 (10.9)	.92
Antenatal steroids, n (%)	23 (82.1)	77 (98.7)	.005*		14 (82.4)	106 (89.1)	.69
Placental abruption, n (%)	2 (7.1)	8 (10.3)	.92		0 (0)	8 (6.7)	.60
Birth weight, M (IQR)	920 (363)	905 (178)	.47		1350 (285)	1280 (250)	.44
Fetal gender (male), n (%)	17 (60.7)	41 (52.6)	.46		12 (70.6)	56 (47.1)	.07
Apgar score < 5 at 5 min, n (%)	7 (25.0)	10 (12.8)	.23		1 (5.9)	12 (10.1)	.91
Presentation at the time of delivery							
Vertex, n (%)	20 (71.4)	57 (73.1)			15 (88.2)	82 (68.9)	
Nonvertex, n (%)	8 (28.6)	21 (26.9)	.87		2 (11.8)	37 (31.1)	.17

BMI = body mass index, IQR = interquartile range, M = median.

* *P* < .05 compared with vaginal delivery.

**Table 4:** Secondary analysis of mortality and morbidity in subgroups according to the gestational age.

Morbidity and mortality	Vaginal delivery, n (%)	Cesarean section, n (%)	Unadjusted OR (95% CI)	Adjusted OR (95% CI)	*P* value
24–27 wk					
Composite neonatal morbidity	27 (96.4)	70 (89.7)	0.32 (0.04–2.72)	0.06 (0.01–1.75)	.10
Death within 28 d of delivery	8 (28.6)	13 (16.7)	0.50 (0.18–1.38)	0.78 (0.21–2.86)	.71
28–31 wk					
Composite neonatal morbidity	11 (64.7)	56 (47.1)	0.49 (0.17–1.40)	0.43 (0.13–1.36)	.15
Death within 28 d of delivery	1 (5.9)	1 (0.8%)	0.14 (0.01–2.28)	0.11 (0.01–2.81)	.18

CI = confidence interval, OR = odds ratio.

## 4. Discussion

The optimal mode of delivery for VLBW infants remains a matter of debate. There have been considerable efforts in reducing morbidity and mortality of VLBW infants in the recent years.^[4]^ Obstetrician´s decision on the delivery plan depends on reliable evidence. In this study, we aimed at investigating the optimal mode of delivery in a Chinese cohort of VLBW infants. To our knowledge, no similar studies have been previously conducted in China.

The CD rate of VLBW infants delivered at 24 to 31 weeks of gestation was similar to that observed in Korea^[8]^ but higher than the one observed in the United States.^[6]^ In our subgroups, the composite neonatal morbidity and mortality within 28 days of delivery were higher compared to those reported in other studies conducted in developed countries,^[6,8,21]^ presumably due to different definitions of neonatal morbidity and our neonatal technology limitations.

Compared with the VD group, a significantly lower composite neonatal morbidity was observed in the CD group. The difference was statistically significant after adjusting for birthweight, twin pregnancy and antenatal steroids intake. Of note, the same difference was not observed in subgroups stratified by gestational age. The most probable interpretation is that gestational age was strongly associated with morbidity in VLBW infants despite we adjusted for birthweight in the multivariate analysis. Another possible reason is that the sample size was not large enough.

In a European population-based cohort study, Schmidt et al^[22]^ found protective effects of CD for very preterm breech infants. Another study^[6]^ conducted in singleton pregnancies between 22 and 29 gestational weeks found that CD reduced neonatal death within 24 hours of delivery but did not affect the overall morbidity and mortality. In a nationwide prospective cohort study, Kim et al^[8]^ found no association among CD, morbidity and mortality of VLBW infants between 23 and 34 gestational weeks. The inconsistencies between our study and prior studies may be due to different sample sizes and study protocols.

The gestational age under 26 weeks was previously associated with an increased risk of severe maternal morbidity in women having a preterm CD.^[23]^ The severe maternal morbidity included future reproductive failures,^[24]^ anxiety and depression.^[25]^ In our study, we did not find a significant association between severe maternal morbidity and preterm CD, presumably due to an inadequate sample size or missing data.

There are several limitations to this study. Firstly, the sample size was relatively small as we did not include infants before 2015, when infants under 28 gestational weeks did not receive adequate medical attention and were often abandoned by their parents. Since then, there have been changes in resuscitative capabilities, but preterm birth is still defined as ≥ 28 weeks in China, meaning that parental choices can still influence medical decisions for these infants. To avoid confounding bias, we excluded deliveries before 2015 and those who refused medical treatment, resulting in a small sample size. Secondly, due to policies in China, different neonatal intensive care units (NICUs) have varying levels of diagnosis and treatment for different gestational weeks, particularly under 28 weeks. When in utero transfer is not possible, emergency transfer to a higher level NICU is still necessary after birth. We have not launched a multicenter study because we believe that emergency transfers under different circumstances may cause bias, and our single-center NICU can provide adequate treatment.

Despite these limitations, our study still provides real-world evidence for the discussion on this topic. However, we were unable to evaluate the relationship between individual neonatal morbidity and mode of delivery separately due to the small sample size, so we used composite neonatal morbidity as our outcome. Additionally, we were unable to precisely identify elective and emergency CDs, which may affect the use of antenatal corticosteroids, although we adjusted for this factor in the multivariate analysis. When communicating with families about delivery methods, our obstetricians consider both neonatal prognosis and maternal complications. We will continue to accumulate sample size to further analyze the impact of different delivery modes on neonatal short- and long-term complications, providing useful evidence for clinical decision-making. Further studies are needed to confirm whether CD alleviates mortality and morbidity in VLBW infants.

## 5. Conclusion

Compared to VD, CD was associated with a lower morbidity in VLBW infants. Additional evidence is necessary to confirm this association and its relationship with gestational age.

## Acknowledgments

We acknowledge TopEdit LLC for the linguistic editing and proofreading during the preparation of this manuscript. And we would like to express our gratitude to Professor Zheng, a biostatistician from Zhejiang Chinese Medical University, who provided statistical expertise and consultation during the preparation of this manuscript.

## Author contributions

**Conceptualization:** Jianqiong Li.

**Data curation:** Jianqiong Li, Gu Liu, Yuyang Chen, Yichao Tian.

**Formal analysis:** Tingting Zhu, Yuyang Chen.

**Project administration:** Fengbing Liang.

**Supervision:** Tingting Zhu, Fengbing Liang.

**Writing – original draft:** Jianqiong Li.

**Writing – review & editing:** Linli Xing, Fengbing Liang.
